# Supplementary figures and images for: dRTEL1 is essential for the maintenance of Drosophila male germline stem cells
Source: PLoS Genet. 2021 Oct 13;17(10):e1009834. doi: 10.1371/journal.pgen.1009834 (PMC8513875; doi:10.1371/journal.pgen.1009834)

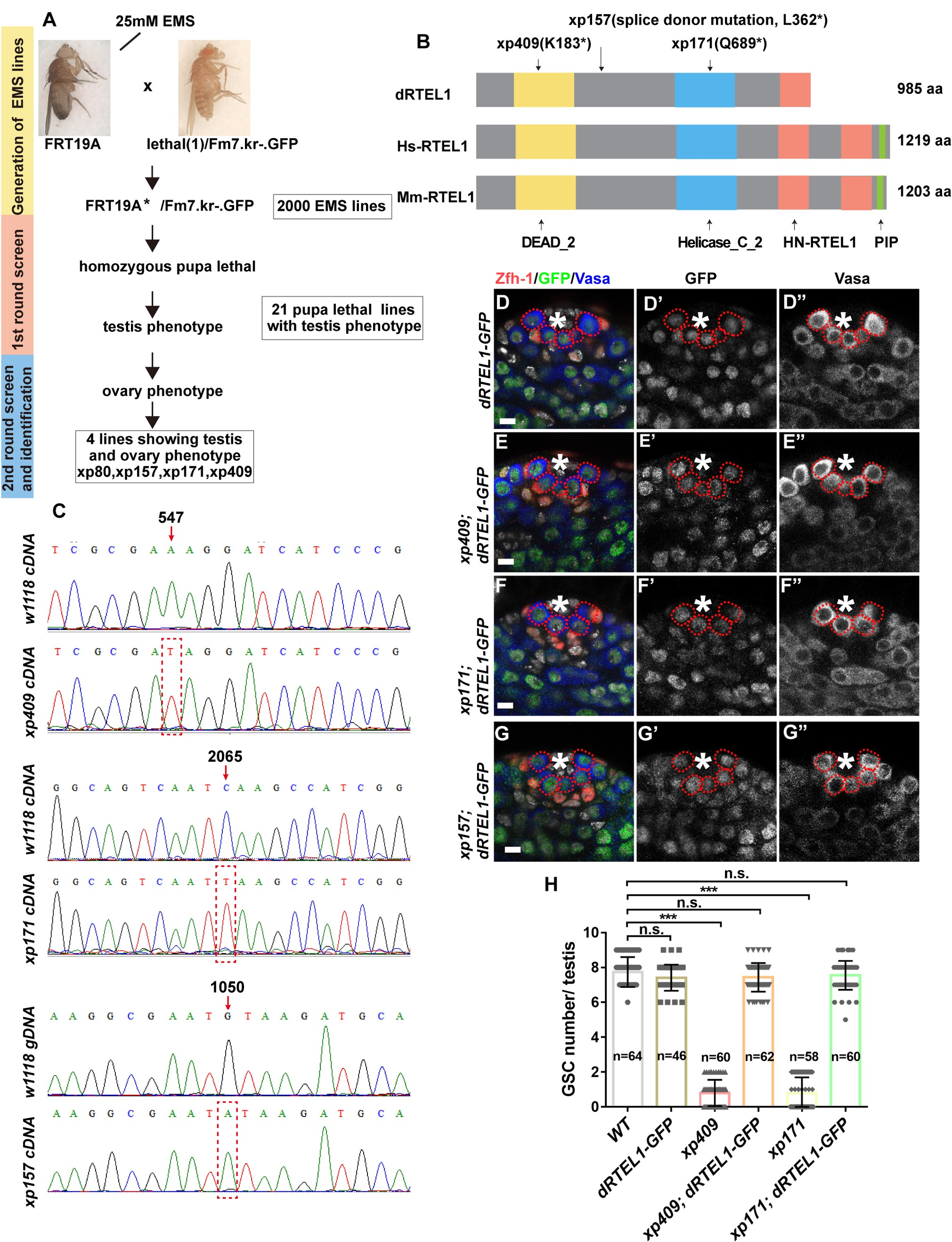

Supplement: S1 Fig — (A) scheme showing EMS-based mutagenesis screening on X-chromosome. FRT19A male flies treated with 25mM EMS and crossed with an X chromosome balancer lethal(1)/Fm7.Kr.GFP to generate about 2000 stocks. After screening these lines, 4 mutant lines identified with defects in both female and male germlines. (B) The protein structure of RTEL1 and the molecular lesion present in the dRTEL1 mutant alleles. Drosophila, human, and M. musculus RTEL1 proteins contain a DEAD_2 (yellow), a Helicase_C_2 (blue) domain and HN_RTEL1 domain (orange), while human, and M. musculus RTEL1 proteins contain an extra PIP box (green). d, Drosophila melanogaster; Hs, Homo sapiens; Mm, Mus musculus. (C) Verification of corresponding mutation in xp409, xp171 and xp157 by RT-PCR followed by sequencing. (D-G”) dRTEL1–GFP (D-D”) transgene fully rescues germline defect observed in xp409 (E-E”), xp171 (F-F”) and xp157 (G-G”) larval gonad at 96 hr ALH. (H) Quantification of the GSC number per testis in various backgrounds. Number in each bar represents the number of testes examined. Data are mean ± s.e. n.s., not significant, *, P<0.05, **, P<0.01, ***, P<0.001. The hub is indicated by asterisks. GSCs are indicated by red dotted circles. DNA (TO-PRO-3) is in white in D-G. Scale bar 5 um. (TIF) [file pgen.1009834.s001.tif]

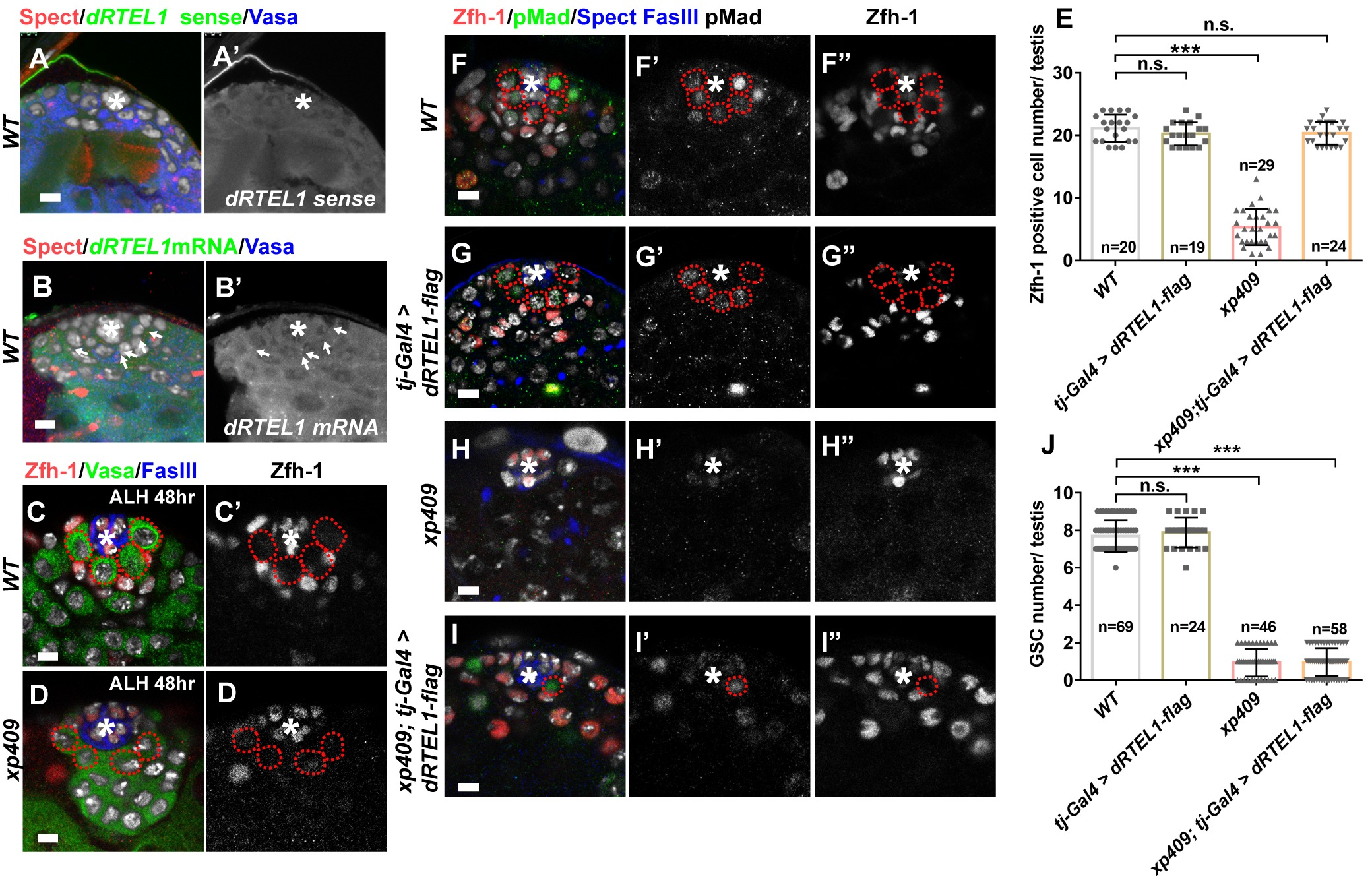

Supplement: S2 Fig — (A) dRTEL1 sense probe detecting no specific signal in WT testis at 96 hr ALH. (B) A WT testis at 96 hr ALH showing dRTEL1 transcripts detected by anti-sense probe (arrows). (C-D’) xp409 testis at 48 hr ALH (D,D’) exhibiting a decrease of somatic cell number compared with WT counterparts (C,C’). (E) Quantification of the Zfh-1 positive cell number per testis in various backgrounds. Number in each bar represents the number of testes examined. (F-I) Representative images of 96 hr ALH testis of WT (F), tj-Gal4 > UAS-dRTEL1-flag (G), xp409 (H) and xp409;tj-Gal4 > UAS-dRTEL1-flag (I). (J) Quantification of GSC number per testis in various backgrounds. Number in each bar represents the number of testes examined. Data are mean ± s.e. n.s., not significant, *, P<0.05, **, P<0.01, ***, P<0.001. The hub is indicated by asterisks. GSCs are indicated by red dotted circles. DNA(TO-PRO-3) is in white. Scale bar: 5 μm. (TIF) [file pgen.1009834.s002.tif]

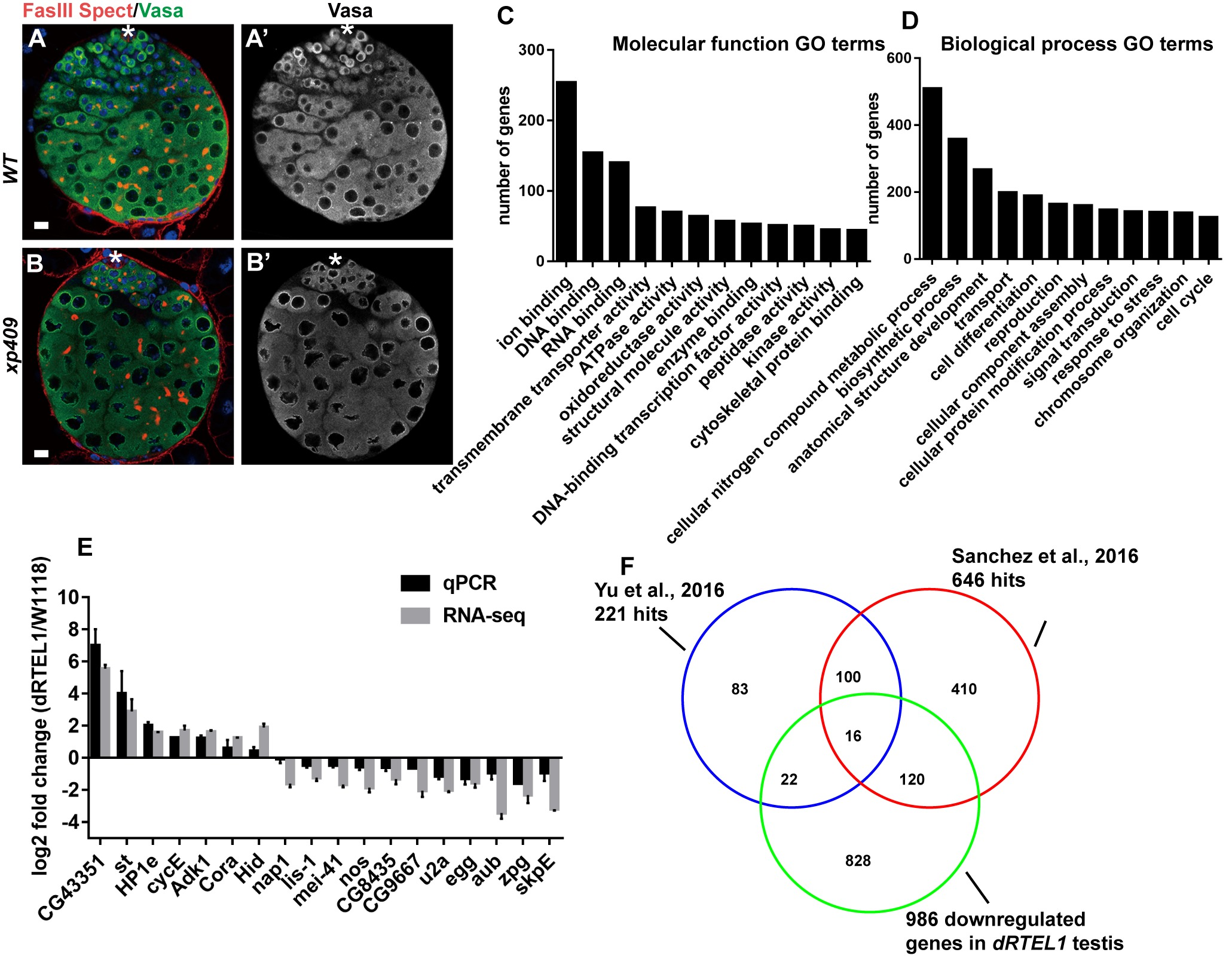

Supplement: S3 Fig — (A-B’) Representative confocal images of GSCs in control (A,A’) and xp409 (B.B’) larval testis at 60–72 hr ALH. ToPro-3 in blue. Scale bar: 10 μm. (C) The differentially expressed genes categorized according to gene molecular function ontology term analysis. (D) The differentially expressed genes categorized according to gene biological process ontology term analysis. (E) The randomly selected genes exhibiting same trend of expression change (by log2 fold) in w1118 testis and dRTEL1 mutant testis in the RNA-Seq (N = 2) and qRT-PCR (N = 3), although the exact fold changes in transcription levels showing some variations. (F) The comparisons of RNA-seq data with 2 published RNAi screens. (TIF) [file pgen.1009834.s003.tif]

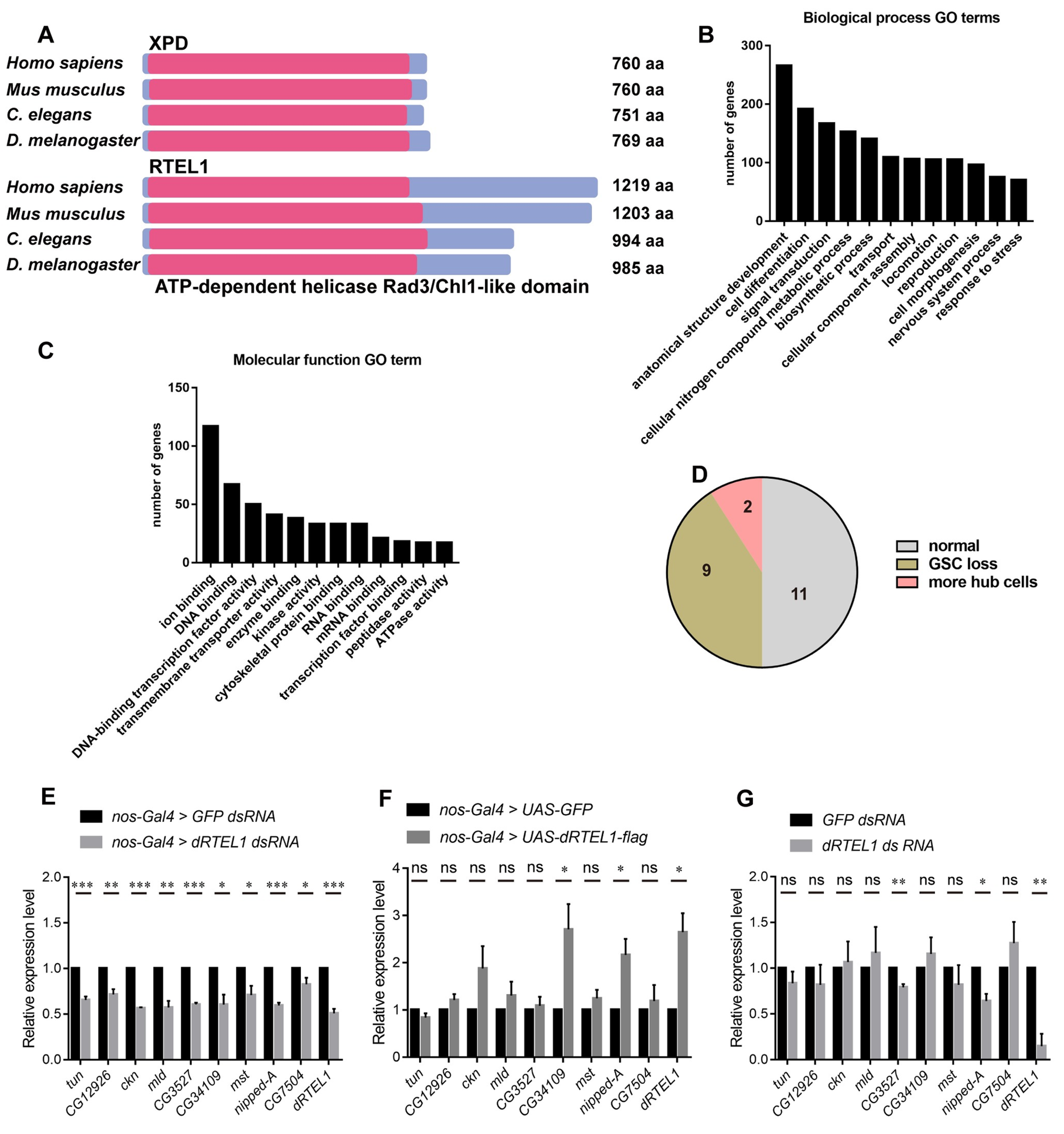

Supplement: S4 Fig — (A) The protein domain structure of dRTEL1 and XPD. (B) GO term (biological process) analysis for genes enriched in dRTEL1 mutant. (C) GO term (molecular function) analysis for genes enriched in dRTEL1 mutant. (D) Summary of a small-scale RNAi screen on the 22 down-regulated overlapping genes in the fly testis. (E) Relative mRNA levels of Nipped-A, mst, CG3527, ckn, mld, CG34109, tun, CG7504, and CG12926 in dRTEL1 germline knockdown testis. (F) Relative mRNA levels of Nipped-A, mst, CG3527, ckn, mld, CG34109, tun, CG7504, and CG12926 in testis with germline overexpression of UASp-dRTEL1-flag in combination with nos-Gal4. (G) Relative mRNA levels of Nipped-A, mst, CG3527, ckn, mld, CG34109, tun, CG7504, and CG12926 in S2 cells with dsRNA-mediated dRTEL1 knockdown. Data are mean±s.e. n.s., not significant, *, P<0.05, **, P<0.01, ***, P<0.001. (TIF) [file pgen.1009834.s004.tif]

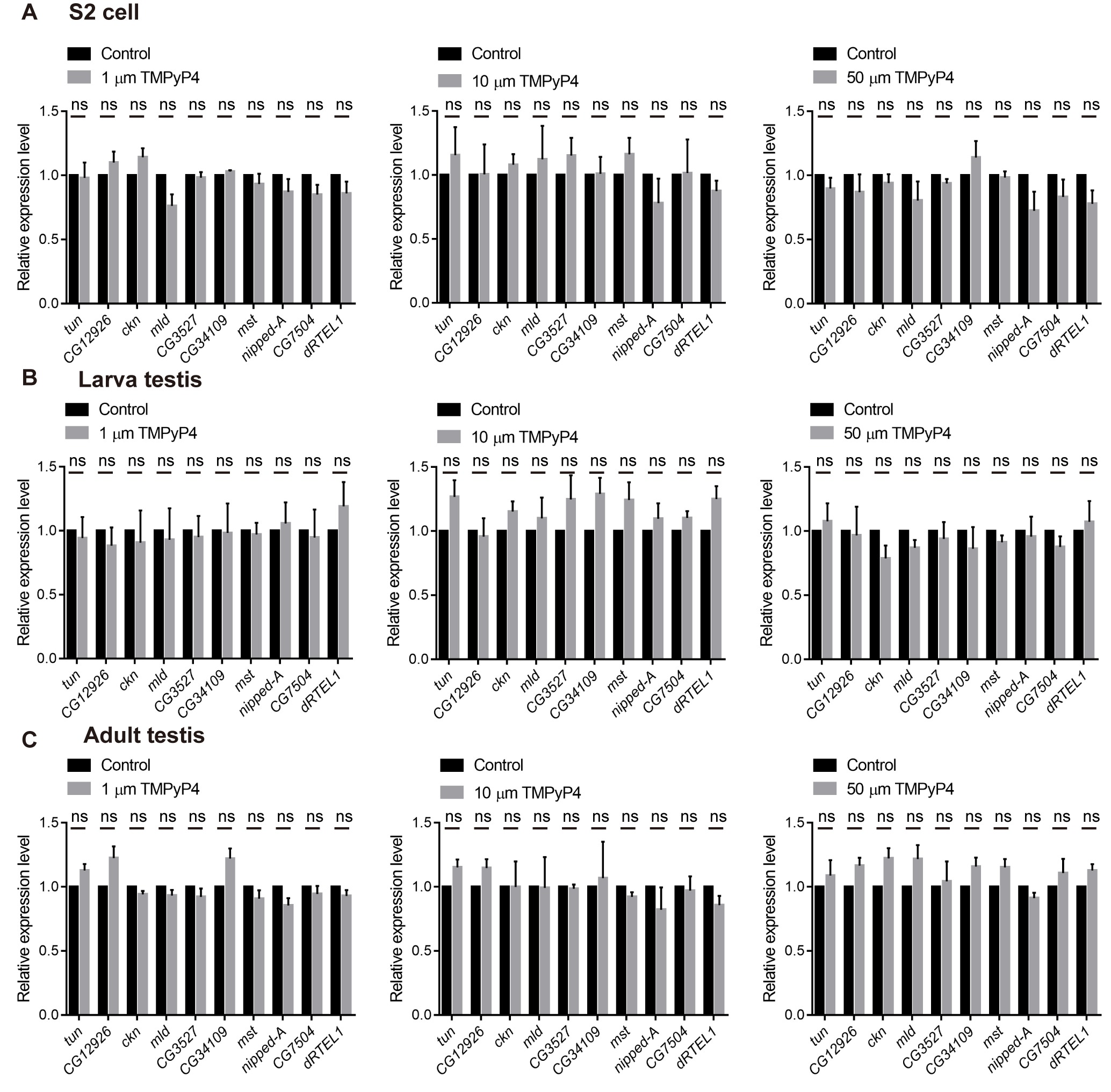

Supplement: S5 Fig — (A) Relative mRNA levels of Nipped-A, mst, CG3527, ckn, mld, CG34109, tun, CG7504, and CG12926 in S2 cells treated with various concentration of TMPyP4 (1 μM,10 μM or 50 μM). (B) Relative mRNA levels of Nipped-A, mst, CG3527, ckn, mld, CG34109, tun, CG7504, and CG12926 in L3 larva testis treated with various concentration of TMPyP4 (1 μM,10 μM or 50 μM) from hatching. (C) Relative mRNA levels of Nipped-A, mst, CG3527, ckn, mld, CG34109, tun, CG7504, and CG12926 in D14 adult testis treated with various concentration of TMPyP4 (1 μM,10 μM or 50 μM) from larval hatching. Data are mean±s.e. n.s., not significant, *, P<0.05, **, P<0.01, ***, P<0.001. (TIF) [file pgen.1009834.s005.tif]

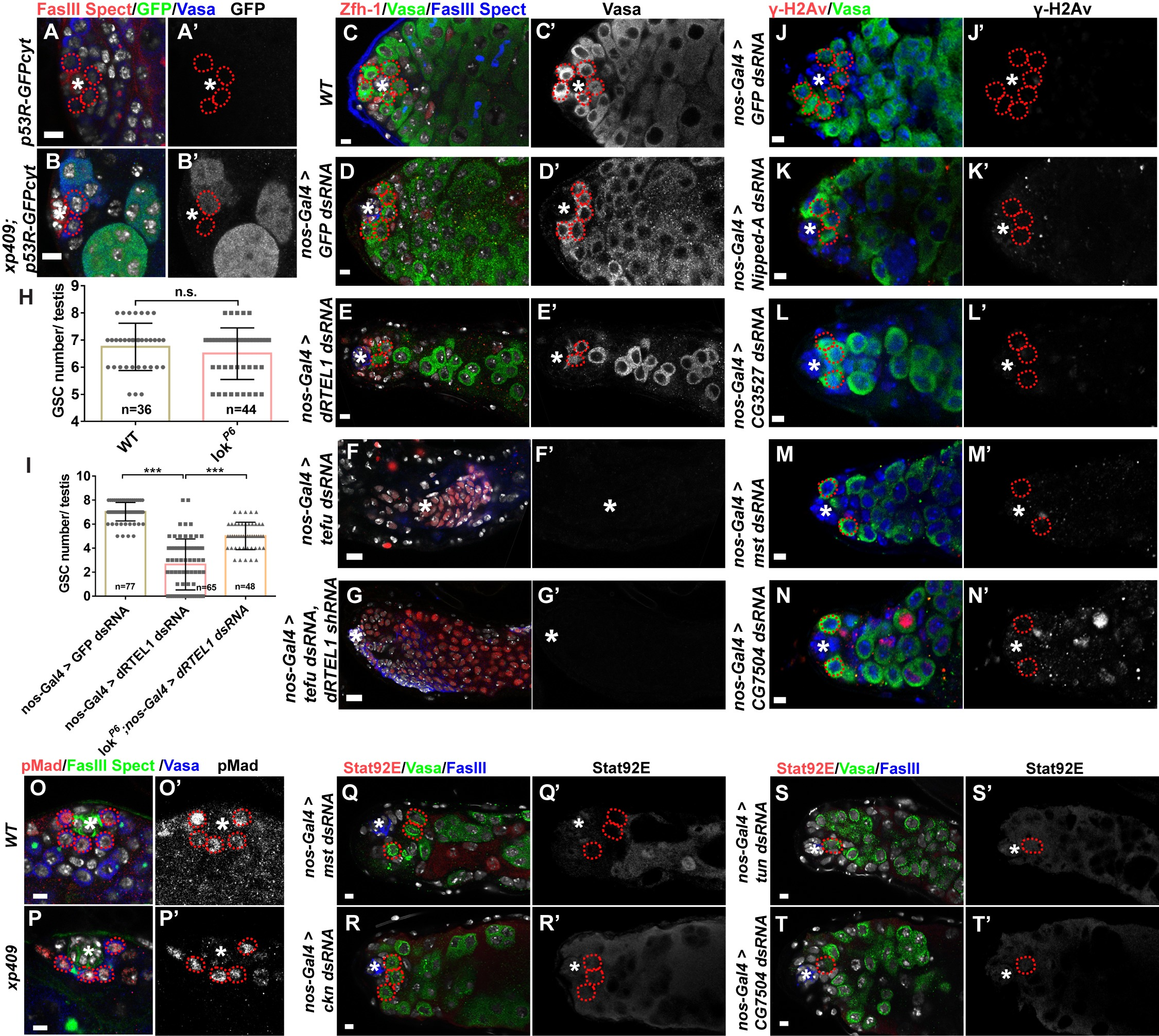

Supplement: S6 Fig — (A,A’) Representative image of p53R-GFPcyt larval testis at 72hr ALH showing no GFP expression in GSCs. (B,B’) A xp409; p53R-GFPcyt larval testis at 72hr ALH showing GFP expression in GSCs. (C,C’) Representative image of D14 WT testis. (D,D′) Representative image of D14 nos-Gal4 > GFP dsRNA testis. (E,E’) Representative image of D14 nos-Gal4 > dRTEL1 dsRNA testis exhibiting GSC loss. (F-G’) Representative image of D1 nos-Gal4 > tefu dsRNA testis (F,F’) or D1 nos-Gal4 > tefu dsRNA, dRTEL1 dsRNA testis (G,G’) showing complete GSC loss. (H) Quantification of GSC number per testis in WT and lokP6. Number in each bar represents the sample number. (I) Quantification of GSC number per testis in various background. Number in each bar represents the number of testes examined. Data are mean±s.e. n.s., not significant, *, P<0.05, **, P<0.01, ***, P<0.001. (J-N’) Representative confocal images of D14 testis showing γ-H2Av expression in nos-Gal4 > GFP dsRNA (J,J’), nos-Gal4 > Nipped-A dsRNA (K,K’), nos-Gal4 > CG3527 dsRNA (L,L’), nos-Gal4 > mst dsRNA (M,M’), and nos-Gal4 > CG7504 dsRNA (N,N’). (O,O’) A WT larval testis at 72 hr ALH showing pMad expression in GSCs. (P,P’) A xp409 larval testis at 72 hr ALH showing pMad expression in GSCs. (Q-T’) Representative confocal images of D14 testis showing Stat92E expression in nos-Gal4 > mst dsRNA (Q,Q’), nos-Gal4 > ckn dsRNA (R,R’), nos-Gal4 > tun dsRNA (S,S’), or nos-Gal4 > CG7504 dsRNA (T,T’). The hub is indicated by asterisks. GSCs are indicated by red dotted circles. DNA(TO-PRO-3) is in white in A-G and O-T and blue in J-N. Scale bars: 5 μm. (TIF) [file pgen.1009834.s006.tif]

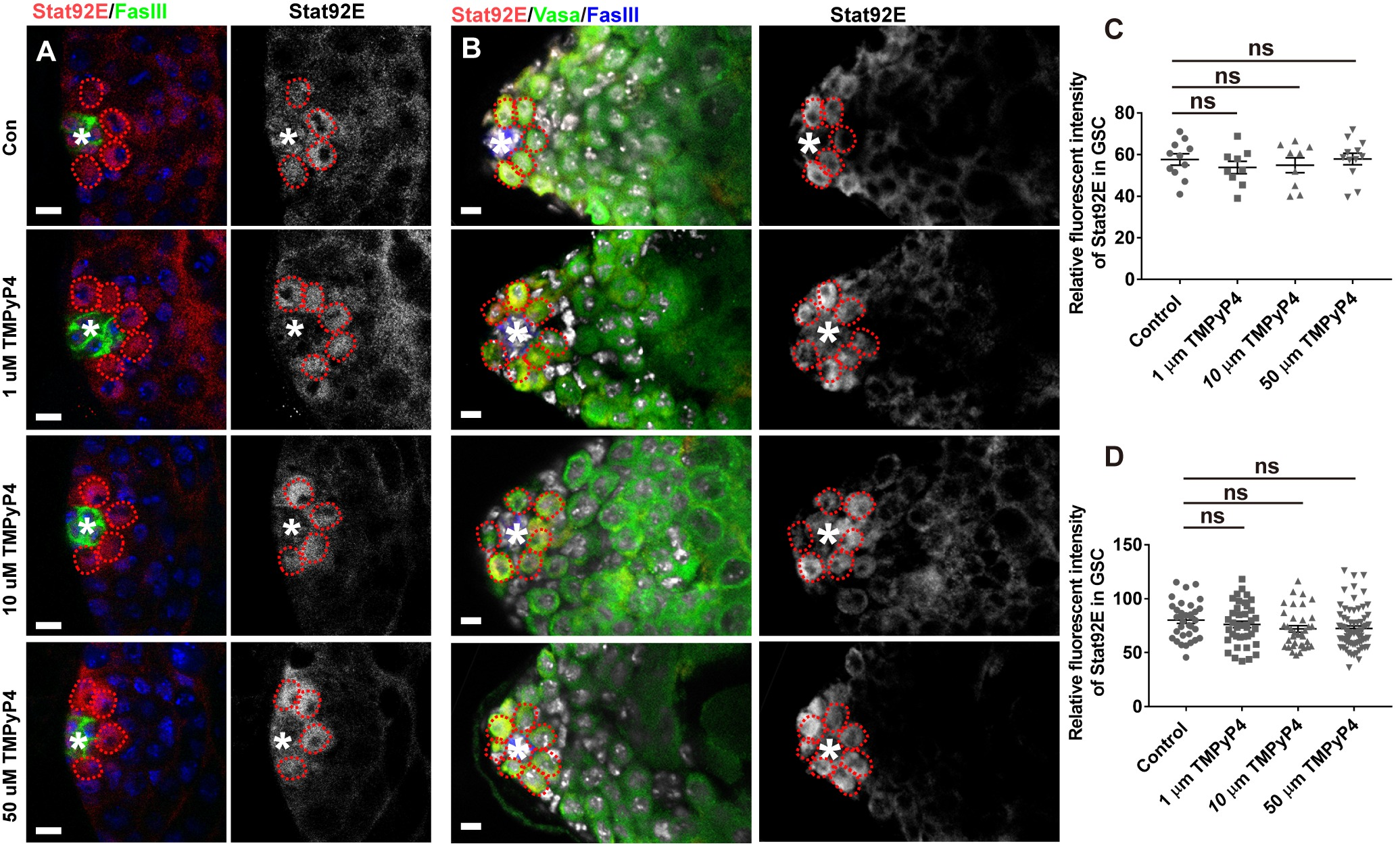

Supplement: S7 Fig — (A) Stat92E expression in WT larval testis (96 hr ALH) treated with 0, 1μM, 10μM and 50 μM TMPyP4. (B) Stat92E expression in WT testis (D14) treated with 0, 1μM, 10μM and 50 μM TMPyP4. (C) Quantification of the relative fluorescent intensity of Stat92E per GSC in larval testis of various backgrounds at 96 hr ALH. (D) Quantification of the relative fluorescent intensity of Stat92E per GSC in various backgrounds at D14. Data are mean±s.e. n.s., not significant, *, P<0.05, **, P<0.01, ***, P<0.001. The hub is indicated by asterisks. GSCs are indicated by red dotted circles. DNA(TO-PRO-3) is in blue in A and white in B. Scale bars: 5 μm. (TIF) [file pgen.1009834.s007.tif]

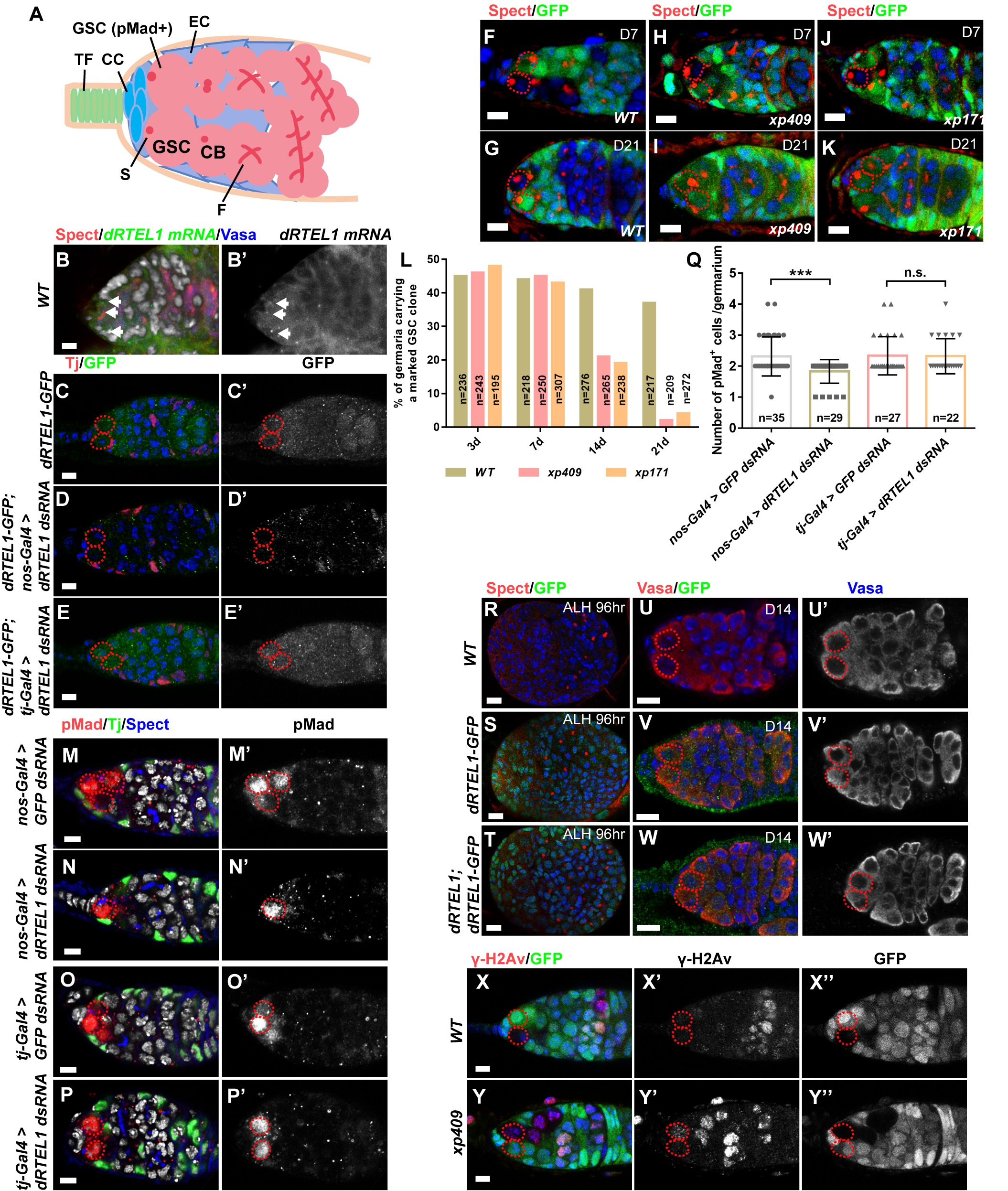

Supplement: S8 Fig — (A) A schematic diagram showing the anterior half of the Drosophila germarium. (B,B’) Representative image of D14 WT germarium showing dRTEL1 transcripts in GSCs detected by anti-sense probe (arrows). (C,C’) Representative image of D14 dRTEL1-GFP germarium showing that GFP expression in germ cells. (D,D’) Representative image of D14 dRTEL1-GFP;nos-Gal4 > dRTEL1 dsRNA germarium showing reduced GFP expression in germ cells. (E,E’) Representative image of D14 dRTEL1-GFP;tj-Gal4 > dRTEL1 dsRNA germarium showing reduced GFP expression in Tj-positive somatic cells. (F-K) Representative images showing a marked GFP-negative GSC clone in control (F,G), xp409 (H,I) or xp171 (J,K) D7 or D21 ACI. Note that D21 xp409 or xp171 germarium do not contain marked GFP-negative GSC clone. (L) Quantification of the percentage of germaria with marked GFP-negative GSC clones in various backgrounds. Number in each bar represents the sample number. (M-P) Representative images of D14 germarium of nos-Gal4 > UAS-GFP dsRNA (M-M’), nos-Gal4 > UAS-dRTEL1 dsRNA (N-N”), tj-Gal4 > UAS-GFP dsRNA (O,O’) or tj-Gal4 > UAS-dRTEL1 dsRNA (P, P’) showing pMad-positive GSCs. (Q) Quantification of pMad positive cells per germarium in H-I. Number in each bar represents the number of testes examined. (R-T) Representative images of ALH 96hr germarium of WT (R), dRTEL1-GFP (S), or dRTEL1; dRTEL1-GFP (T). (U-W) Representative images of D14 germarium of WT (U-U’), dRTEL1-GFP (V-V’), or dRTEL1; dRTEL1-GFP (W-W’). (X-X’) A D14 germarium showing no γ-H2Av detected in marked GFP-negative WT GSC. (Y-Y’) A D14 germarium showing γ-H2Av accumulation in marked GFP-negative dRTEL1 GSC. Data are mean±s.e. n.s., not significant, *, P<0.05, **, P<0.01, ***, P<0.001. GSCs are indicated by red dotted circles. DNA (TO-PRO-3) is in white in B and M-O and blue in C-K and R-Y. Scale bar: 10 μm. (TIF) [file pgen.1009834.s008.tif]

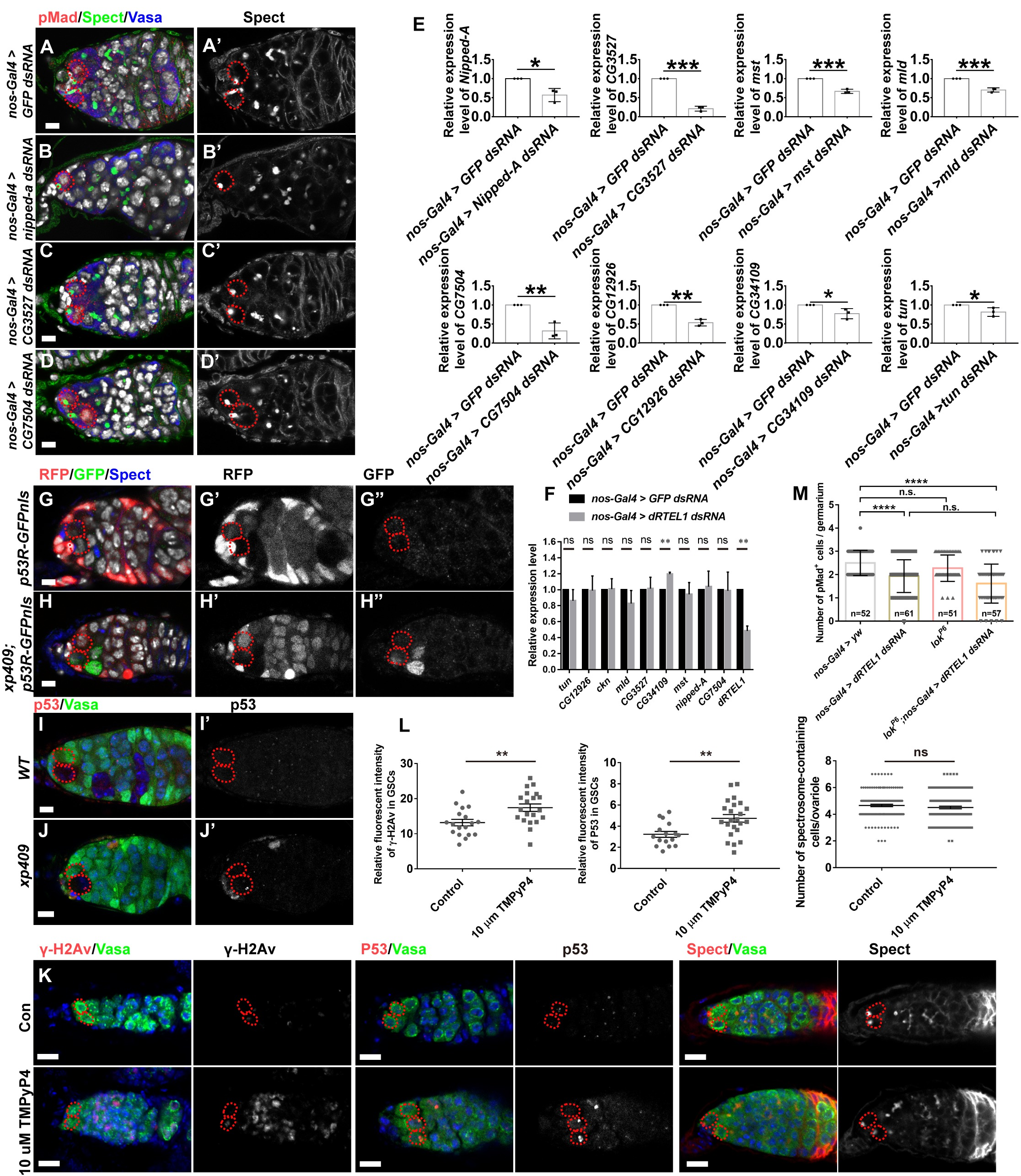

Supplement: S9 Fig — (A,A’) A representative image of D14 nos-Gal4 > UAS-GFP dsRNA germarium. (B,B’) A representative image of D14 nos-Gal4 > Nipped-A dsRNA germarium exhibiting germline tumors filled with pMad-negative, spectrosome-containing undifferentiated germ cells. (C,C’) Representative image of D14 nos-Gal4 > CG3527 dsRNA germarium containing 2 GSCs. (D,D’) Representative image of D14 nos-Gal4 > CG7504 dsRNA germarium containing 2 GSCs. (E) Relative mRNA levels of Nipped-A, mst, CG3527, ckn, mld, CG34109, tun, CG7504, and CG12926 in dRTEL1 germline knockdown ovary. (F) Quantification of the knockdown efficiency in ovary by qPCR. (G-G”) A D14 marked RFP-negative WT GSC does not express p53R-GFPnls. (H-H”) A D14 marked RFP-negative xp409 GSC expressing p53R-GFPnls. (I-I’) A D14 marked GFP-negative WT GSC showing no p53 expression. (J-J’) A D14 marked GFP-negative xp409 GSC showing elevated p53 expression. (K) γ-H2Av and p53 expression in D14 WT ovary treated with 0, 1μM, 10μM and 50 μM TMPyP4. (L) Quantification of the relative fluorescent intensity of γ-H2Av and p53 in GSCs and number of GSCs per ovary in various backgrounds at D14. (M) Quantification of pMad positive cells per germarium in various backgrounds. Number in each bar represents the number of testes examined. Data are mean ± s.e. n.s., not significant, *, P<0.05, **, P<0.01, ***, P<0.001. (L-L’) DNA (TO-PRO-3) is in white in A-D and G-H and blue in I-K. GSCs are indicated by red dotted circles. Scale bars: 10 μm. (TIF) [file pgen.1009834.s009.tif]
