# Supplementary material for: dRTEL1 is essential for the maintenance of Drosophila male germline stem cells
Source: PLoS Genet. 2021 Oct 13;17(10):e1009834. doi: 10.1371/journal.pgen.1009834 (PMC8513875; doi:10.1371/journal.pgen.1009834)
Supplement: Attachment — Submitted filename: PGENETICS-D-21-00484_R1_reviewer#1_comments.pdf [file pgen.1009834.s016.pdf]

The authors provided responses to most of our concerns and the revised version is much improved. Additional experiments explain clearly the independence of DSB and GSC loss through dRTEL1 and the statement became more convincing. For potential publication of this manuscript in PLoS genetics, however, the points raised below (#1~5) need to be further amended and/or addressed.

1) The phenotype of CHK1_grp was similar to the CHK2_lok and well-presented in the revised version. Is CHL1_grp also involved in the Stat 92E activation like CHK2_lok? It will be good to provide some results or argument about this.

Q9: Involvement of the other DDR components (ATM_tefu, CHK1_grp) in the STAT92E mediated GSC maintenance could be addressed. If not, the authors should provide any argument for the abovementioned issue.

We appreciate this reviewer's insightful suggestion. We have examined Stat92E expression in the testis of germline-specific knockdown of *grp* and do not observe any defect in its expression (Fig. 8M), consistent with the observation that these testes do not exhibit a GSC loss phenotype. However, we are not able to examine Stat92E in *tefu* knockdown testis as these larval gonads do not contain any germ cells, suggesting that tefu functions early in development for the formation of GSCs. As discussed in previous concern (#8), our new results show that induced DSBs do not play a major role in the maintenance of male GSCs (Fig. 6 and S7). Our results show that dRTEL1 acts through multiple downstream pathway including expression of target genes and the DDR pathway to maintain fly malel GSCs.

2) mRNAs of those 'target genes' were reduced in the dRTEL1 mutant, and ChIP-Seq reveaed dRTEL1 was bound to their loci. However, no genetic evidence was shown about the interaction of downstream 'target genes' and dRTEL1 in loss of GSC. The authors speculated that the multiple downstream targets would not specify the Stat92E level in their overexpression. How do authors think about this? Do those multiple downstream targets function redundantly for the GSC maintenance? Any argument could be provided for this concern.

Q10: I wonder if overexpression of dETEL1 target genes would recover the defect of dETEL1 that gives STAT92E reduction and GSCs loss. Did the authors perform any experiments addressing this question? Alternatively, any argument could be provided for this concern.

We are sorry that we have not conducted these rescue experiments due to lack of transgenic stocks to overexpress these genes. Our results indicate that dRTEL1 may act through multiple downstream targets to regulate Stat92E level (Fig. 8N). Some of these candidates also affect Stat92E level (Fig. S6Q-T). It is possible that restoring single gene in dRTEL1 mutant might not be able to rescue Stat92E level although we have not tested this.

3) dRTEL1 in female GSCs;

dRTEL1 mutant exhibited GSC loss and p53 upregulation, but no reduction of expression of any target genes in females. Nevertheless, Nippeled-A showed a severe phenotype in ovaries.

3-1) Can dRTEL1 over-expression rescue the defect of GSCs in dRTEL1 mutant female like in testes?

3-2) CHK2_lok doesn't seem to be involved in dRTEL1 regulating GSC loss (Fig9R). In addition, most of the target genes in testes were not relevant to female GSC loss, implying a different mechanism from that in testes. Can the authors interpret or speculate about the importance of dRTEL1 function in females? With this context, it also might be better to explain the further molecular role of Nippeled-A in female GSCs.

3-3) The focus of this study is more about the function of dRTEL1 in male GSCs maintenance, I suggest moving data addressing that in females to supplementary.

4) There is no description of amended Fig8M. Please add.

We have now added these information in the text (page 8-9)

For each ChIP experiment, ~800 males were dissected and testes were pooled together as one sample. For L3 testis, it requires more than 1,000 pairs of larvae testes (according to our experience) to get similar amount of material for one experiment.

5) The above information cannot be found the revised version (page 8-9). Please check.
