# Supplementary material for: dRTEL1 is essential for the maintenance of Drosophila male germline stem cells
Source: PLoS Genet. 2021 Oct 13;17(10):e1009834. doi: 10.1371/journal.pgen.1009834 (PMC8513875; doi:10.1371/journal.pgen.1009834)
Supplement: Attachment — Submitted filename: Response to reviewers.docx [file pgen.1009834.s015.docx]

**Response to reviewer:**

**We thank you for your time and efforts spent reviewing and providing feedback on our manuscript. We have addressed all your comments/concerns point by point. Please note that all changes in relation to your comments/concerns are blue-colored in this revised manuscript. We truly believe that your constructive comments have led to a much-improved manuscript.**

Reviewer #1: Dear Authors,

Yang et al manuscript titled ‘dRTEL1 Is Essential for the Maintenance of Drosophila male Germline Stem Cells’ is a very compelling and comprehensive story indicating a novel function of Drosophila RTEL1 as a regulator of GSCs. This article, once published, will be of great interest to the PLOS Genetics audience.

The authors utilized a number of different techniques to address their research questions. In the paper we can see the mastery of confocal imaging and traditional genetic approaches like gene knockdown and rescue, complemented by big data analyses (ChIP-seq and RNA-seq). RTEL1 helicase, traditionally implicated in telomere maintenance and DNA repair, as demonstrated here, does seem to play a critical role in germline stem cell maintenance. The authors thoroughly investigate RTEL1’s role in Drosophila testis GSCs, however a brief look at the female germline indicates some elements of this regulation are conserved which present an opportunity for a follow-up publication.

I did not find any major issues preventing the publication of this manuscript (perhaps with the exception of a few missing controls). However, there is a number of minor revisions the authors should address to help the Plos Genetics audience fully appreciate the scope, quality, and significance of this paper:

**We thank this reviewer for his/her positive comments on our study.**

Data presentation:

1) To facilitate the identification of GSCs, the authors had an excellent idea to provide a schematic of testis in Figure 1A and mark the hub cells with an asterisk throughout the paper. For the benefit of the audience that is not versed in the testis topology, I would strongly suggest outlining GSCs (and other cells which the phenotype the authors discuss or compare) in all images. The authors already demonstrated the importance of this approach in Fig 8M-N where the cells of interest are outlined. Figure 8 could also benefit from a germarium schematic alike the one seen in Figure 1A. In figures discussing subcellular structures e.g., Figure 3K, arrows could be used in a similar fashion to how the data is presented in Figure 3L, which would improve the reading experience.

**We thank this reviewer for pointing this out and have now added outlines for GSCs in all images. We also add a schematic of germarium in Fig. 9A and arrows in Fig. 3R (Fig. 3K in previous submission).**

2) In line 134 the authors explain the significance of α-Spectrin and Vasa stains. With the exception of Figure 4, all figures contain images of cells stained with either Zfh-1, pMad, or FasIII. Zfh-1 is mentioned once in line 166, but the significance and the role of this stain needs to be elucidated. Similarly, pMad is mentioned for the first time in line 355 in the context of Figure 7. However, the readers could benefit from knowing the significance of this stain much earlier in the story since pMad is labeled in Figures 2+. I did not find any mention of FasIII in the manuscript. The information regarding FasIII should be added to the Materials and methods section (line 528) as well. The schematic in Figure 1A presents an excellent opportunity to introduce all these stains to the audience as well.

**As the reviewer suggests, we have now introduced all these markers at the earlier section of our manuscript. pMad information is added in “Introduction” section (see page 5, line 76-80). All makers mentioned in this study including pMad, FasIII and Zfh1 is now summiarized in Figure 1A. The anti-FasIII antibody information is also added in “Materials and Methods” section (page 33, line 687-688).**

3) The authors in line 131 mention ‘a typical Drosophila larval testis contains 6 to 9 GSCs adjacent to the hub located at the apical tip of the testis’. The schematics in Figure 1A contains 3 GSC cells while the average GSC count per testis in Figure 1H is almost 8. Does the manuscript contain any images where more than 5-6 GSCs are present? Perhaps a clarification is needed in the ‘Quantification and Statistical analysis’ (line 681), whether the missing GSCs are on the distal side of the testis, therefore hidden from sight? Should it be the case, how are these quantified? Do the images used for quantification look like Figure 3A or 3K, and the lateral view images like Figure 3B, 3L, 3M are excluded?

**We are sorry for not explicitly describing GSC counting method in previous submission. As this reviewer mentioned, there are fewer GSCs in those confocal images because some GSCs are not in the focal plane and thus are not seen in these confocal images. GSCs were counted under a fluorescent microscope using the criteria described, not from those confocal images. We have now clarified the quantification method in the ‘Quantification and Statistical analysis’ of “Materials and Methods” section (page 39) as follow:**

***Number of GSCs or spectrosome-containing cells were counted from randomly selected testes or ovaries under a fluorescence microscope and every GSC was carefully assessed by adjusting the focus to include all GSCs.***

4) In the section concerning female GSCs (line 385), the authors indicated they are counting the GFP-marked GSCs (line392-394). The audience could benefit from a note on how these cells were marked. Additionally, In Figure 8G the authors perform a quantification of the % of germaria carrying a marked GSC clone. Figure 8A needs a series of images that depict both GFP-marked and GFP-lost cells to convey how do the quantified phenotypes look like with the cells of interest outlined. Additionally, the Figure 8 legend (lines 1057-1058) contains a reference to the RFP signal. The audience could benefit from a thorough description of how the RFP signal came into play and its significance.

**We thank this reviewer for pointing this out. In these experiments, we deployed two different fluorescent markers (GFP and RFP) to mark *dRTEL1* mutant GSCs. For Fig. 9B-G (Fig. 8B-G in previous submission), Fig. 9N-O and S8K-L, female *dRTEL1* mutant clones were generated using FLP-mediated mitotic recombination technique using GFP as a marker.** **The mutant GSC is marked by loss of GFP signal, while control GSC expresses the GFP marker. GFP-marked and GFP-lost GSCs are now outlined with red dot circles in Fig. 9 and Fig. S8. For Fig. 9P-Q (Fig. 8N-O in previous submission), we used FLP-mediated mitotic recombination technique to generate clones coupling RFP as a marker because p53R-GFPnls carries a GFP biosensor. In both experiments, marked GSCs were counted under a fluorescent microscope, similar to the GSC counting method for male GSCs. We have now added these information in figure legend and clarified the GFP and RFP signal in “Materials and Methods” (page 32 and 39) as follow:**

***Flies with genotype of FRT19A. ubi-GFP; hs-flp (*Fig. 9B-G, Fig. 9N-O and S8K-L), *or FRT19A.ubi-RFP;hs-flp* (Fig. 9P-Q) *was crossed with flies with genotype FRT19A. xp409, FRT19A. xp171, or FRT19A. xp157 to generate fluorescent signal marked (negative) GSC clones.***

***The % of germaria carrying a marked GSC clone was calculated by dividing the number of germaria carrying GFP-negative marked or RFP-negative marked GSC clone by the total number of germaria checked.***

5) The authors developed and described the nature of 3 dRTEL1 mutants xp157, xp171 and xp409 (lines 113-130). Traditionally, a Western Blot approach would be utilized to inform whether the protein of interest is being produced (either full-length, a truncated form, or no protein at all). It would be too much to ask to conduct such analysis given the lack of commercially available dRTEL1 antibodies, however, these mutants could be characterized further at the mRNA level to confirm the sequence-based predictions.

**We thank this reviewer for pointing this out and have now conducted RT-PCR to amplify the predicted region from these mutants followed by DNA sequencing. These results, as shown in Figure S1C, confirmed these respective mutations.**

6) In Figure 3I-J, the authors performed a propidium iodide staining to infer regarding cell-death in dRTEL1 deficient cells. Negative results without a positive control are impossible to interpret. Are the cells propidium iodide negative because they have an intact membrane, the imaging conditions were suboptimal, or a different reagent was accidentally used? The authors should include a condition in which the audience of the paper can see how propidium iodide-positive testis cells look like. Similarly Figure 3A-B requires a positive control for Caspase-3 staining and Figure 3C-D requires a positive control for TUNEL (these images could become a part of the supplementary material but are necessary for proper data interpretation). It is worth noting that all xp409 images, unlike WT, present a weak signal in these 3 assays. Is it a background or is it real? The interpretation of this section could change depending on what the positive controls look like.

**We thank this reviewer for pointing this out. Regarding PI, TUNEL and cleaved-Caspase 3 stainings, we do not detect expression of these cell death markers in GSCs of both *WT* and *dRTEL1* mutant testes, which leads us to conclude that the loss of GSCs in dRTEL1 mutant is not a result of cell death. As we presented in previous submission, we are not able to detect these markers is GSCs under our experimental condition, suggesting GSCs are more resilent to cell death. Using an induced cell death background by ectopic expression of cell death inducer hid, we are able to detect the expression of these markers in germ cell cysts in larval testis, showing that these detection methods work in these tissues. We have now added these positive controls in Fig 3 (3C for cleave-Caspase-3 staining, 3F for TUNEL staining and 3M for PI staining).**

7) Line 364 reads ‘We noted that the levels of Stat92E protein decreased strongly in dRTEL1 larval testes (Fig.7B).’ Quantification and clarification might be needed regarding what constitutes a ‘strong decrease’. Given the signal spans the entire testis, what is the area of interest? Since dRTEL1 mutants exhibit diminished number of GSCs could the loss of Stat92E signal be attributed to the loss of GSCs instead?

**We thank this reviewer for pointing this out. The Stat92E level is measured for GSCs. In *WT*,** **Stat92E is enriched in GSCs and some of their immediate daughter cells, called gonialblasts (GBs), but rarely detected in differentiated germ cells. For these experiments, Stat92E level is measured for those cells showing GSC characters (containing a spectrosome, Stat92E-positive and next to the hub). Thus, the reduced Stat92E signal is not attributed to the loss of GSCs. The quantification for Fig. 8A-B (Fig. 7A-B in early submission) data is shown in Fig. 8C (Fig.7P in previous submission). We have also clarified this information in the Fig. 8C and 8J by changing Y-axis title to “Relative Fluoroscence intensity of Stat92E in GSCs”.**

Reviewer #2: The manuscript entitled as dRTEL1 Is Essential for the Maintenance of Drosophila male Germline Stem Cells by Yang et al. describes function of CG4078. CG4078 encodes a fly homolog of Regulator of Telomere Elongation Helicase and paralogue of XPD. Though dRTEL is expressed in germ and somatic cells, the only germline expression contributes GSCs maintenance cell-autonomously in males. GSCs loss is not due to apoptosis but precocious GSC differentiation. ChIP and genetic analyses identified 9 functional regulated genes including Nipped-A those cause GSC loss. Curiously, GSCs maintenance by dRTEL was independent of Dpp pathway but dependent on the JAK-STAT pathway. dRTEL1 was also required in the female germline to maintain ovarian GSCs to prevent the activation of the DDR. However, the downstream genes and molecular mechanisms are yet to be identified. Though dRTEL’s primary and/or direct target(s) triggering precocious differentiation resulting in GSC loss remains elusive, comprehensive analyses and convincingly demonstrated the important role of dRTEL. The contents of this study are suitable to be published in PLoS genetics. The authors should address concerns and points described below, making the results more convincing and figures appropriately organized. Especially, the analyses of mRNA-seq and ChIP-seq should be described carefully in the main text and method section. Second rounds of revision would be necessary.

**We thank this reviewer for his/her positive positive comments.**

Major points:

1. As to manuscript organization: mutant alleles and knockdown animals at different stages, such as larva and adult, were examined in each experiment. To avoid readers’ confusion, the stage of the examined samples should be clearly described in both main figs and supplemental figs.

**We thank this reviewer for pointing this out and have now described the stages of examined samples in their respective figures and supplementary figures.**

2. L208-218: dRTEL1 mutant testes exhibited precocious differentiation of germline cells. It would be more convincing if the authors identify spermatogonia by any specific markers, such as Stat92E, in addition to spectrin staining.

**We thank this reviewer for pointing this out and have now added the GSC marker Stat92E (Fig. 3P, 3Q, 3T and 3U).**

3. Line 219: the mRNA-seq data may reflect the loss of germline cells in dRTEL1, not the change of mRNA levels, because the GSCs were lost in mutant ALH 96 hr testes. ALH 60-72 hr testes were used as the RNA-seq samples without any explanation. The GSC numbers and the staining images should be shown at ALH 60-72 hr, or this concern should be described in the main text.

**As presented in Fig. 1, *dRTEL1* testis initially hosted 6-8 GSCs at 48 hr ALH (Fig. 1C, 1D, and 1H), similar to *WT* control. However, GSCs were gradually lost during larval development and by 96 hr ALH. *dRTEL1* testis contained fewer GSCs (Fig. 1F-1H). To minimize the difference between control and *dRTEL1* samples and compare the transciptome difference between *dRTEL1* mutant and *WT* germ cells, we collected early (60-72hr) L3 testis when *dRTEL1* testes still contained many GSCs and GBs. We have now added the repsentative testis images in Fig. S3A-B and explained this in main text (page 12, line 233-235) as follow:**

**The samples were collected from early (60-72hr) L3 testes when *dRTEL1* testes still contained many GSCs and GBs to minimize the difference between control and *dRTEL1* testes (Fig. S3A and S3B).**

4. Line 237: ‘Among those 986 down-regulated genes in dRTEL1 testes, 136 genes were previously identified…’ Is there any reason to focus on the down-regulated genes? Among 831 up-regulated genes in dRTEL1 testes, how many genes are included in the previous studies cited here?

**We focused our analysis on those down-regulated genes because that several genome-wide germline knockdown screens have been conducted in fly ovary and testis and identified a list of candidate genes required for the maintenance of male or female GSCs. These provide a base for us to identify candidate genes essential for GSC maintenance. It is also possible that ectopic expression of some cadidates among 831 up-regulated genes also contribute to GSC loss in dRTEL1 mutant. However, we did not pursue this direction because there is no genome-wide/large scale over-expression screen has been conducted. We have now added this information in the text (see Page 13, line 235-257).**

5. Line 242: ‘These data show that dRTEL1 regulates genes required for GSC maintenance, directly or indirectly.’ This sentence may be overemphasized. There are only 20% overlap between the listed genes in this study and those in the previous studies. Thus, 80% genes are not overlapping. Is there any trend that overlapping genes have higher score or non-overlapping genes show lower score in mRNA-seq analysis performed in this manuscript?

**We appreciate this reviewer pointing this out. We have now removed this sentence. We do not find any trend for the over overlapping genes and non-overlapping genes in terms of higher/lower score in mRNA-seq analysis.**

6. Line 256: regarding to the normalization, more details of the ChIP-seq protocol should be added in text and method sections. How was the normalization performed?

**We thank this reviewer for pointing this out. The RNA-seq and ChIP-seq and analyses were outsourced to BGI (Beijing Genomics Institute, Shenzhen, Guangdong, China). We have now requested this information from BGI and added the analysis methods in “Materials and Methods” as fellow:**

**“ChIP-seq libraries following the manufacturer's instruction. The Illumina compatible libraries were sequenced with Mi-seq desktop sequencer (Mi-Seq, Illumina) by BGI, China. 75 bp single-end read sequencing was accomplished. After sequencing data was delivered, data filtering including removing adaptor sequences, contamination and low-quality reads from raw reads was conducted. After filtering, the remaining reads were called "clean reads", which were mapped to Drosophila genome (dm6, FlyBase 6.05). Then the alignment results were used to calling peak. The model-based analysis of ChIP-Seq (MACS) peak-finding algorithm version 1.4.1 was used for peak calling. The candidate Peak region was extended to be long enough for modeling. Dynamic Possion Distribution was used to calculated p-value of the specific region based on the unique mapped reads. The region would be defined as a Peak when p-value < le-05. Later, the peak information was used for standard bioinformatics analysis. MAnorm was used to identify differential peaks between samples. The log2 ratio of read density between samples M was plotted against the average log2 read density A for all peaks, and robust linear regression was applied to fit the global dependence between the M-A values of common peaks. Then the derived linear model was used as a reference for normalization and extra polated toall peaks. Finally, the P-value for each Peak was calculated based on Bayesian model, the significant regions were picked up if |M| >= 1 and p-value<=10-5. ”**

7. Line 256: ‘dRTEL1-GFP was found to be enriched at 654 genomic loci’ What the criteria to pick the genomic loci from the MACS2 output? The MACS2 output file would be helpful for readers if added in the supplementary. In addition, it should be mentioned in the main text that dRTEL1-GFP expressed not only in GSCs, but in both germline cells and somatic cells.

**These analyses were also conducted by BGI. The model-based analysis of ChIP-Seq (MACS) peak-finding algorithm version 1.4.1 was used for peak calling. The candidate Peak region was extended to be long enough for modeling. Dynamic Possion Distribution was used to calculated p-value of the specific region based on the unique mapped reads. The region would be defined as a Peak when p-value < le-05.**

**As we metioned in the “Materials and Methods”, the CHIP-seq Data including MACS output file generated during this study are available at Mendeley Data (**

[**https://data.mendeley.com/datasets/vyzccs8tsh/1**](https://data.mendeley.com/datasets/vyzccs8tsh/1)**).**

**The expression pattern of dRTEL1-GFP is stated in page 9, line 159-162, as fellow: “Consistently, immunofluorescence analysis of testis carrying a functional dRTEL1-GFP transgene revealed that dRTEL1–GFP expressed in both somatic cells (including CySCs and cyst cells) and germ cells (including GSCs and GBs) (Fig. 2A for adult testis and S1D for larval testis).”**

8. H2Av should be examined in ATM_tefu and CHK1_grp mutants as shown in CHK2_loki.　In addition, p53 should be examined in CHK2_loki and the double KD/KO with dRTEL1. The epistatic hierarchy of DNA damage and DDR pathway genes can be more refined, by examining other components (ATM_tefu, CHK1_grp) of DDR pathway as well.

**We have now carried out experiments to examine H2A and p53 stainings in these suggested components of the DDR pathway. Our results show that knocking down CHK 1_grp activity in male GSCs does not lead to change in expression of H2Av and p53 (Fig. 7E and 7X), in line with our observation that grp is dispensible for male GSC maintenance. We are not able to address these markers in ATM_tefu knockdown GSCs because these larval gonads do not contain any GSC, indicating that tefu is essential for the formation of GSC. Regarding p53 staining in CHK2_loki and *loki;dRTEL1* double mutant, our results show that p53 is not upregulated in *loki* mutant (Fig. 7G, 7I), but is upregulated in double *loki;dRTEL1* double mutant (Fig. 7G, 7I, 7J), similar to H2Av expression in these backgrounds (Fig. 7A-7D). Our new results (Fig. 6 and S7) provide an unexpected data showing that DSBs (presumably its downstream the DDR pathway) likely do not play an essential role in the maintenance of fly male GSCs (Fig. 6 and S7).**

9. Involvement of the other DDR components (ATM_tefu, CHK1_grp) in the STAT92E mediated GSC maintenance could be addressed. If not, the authors should provide any argument for the abovementioned issue.

**We appreciate this reviewer’s insightful suggestion. We have examined Stat92E expression in the testis of germline-specific knockdown of *grp* and do not observe any defect in its expression (Fig. 8M), consistent with the observation that these testes do not exhbit a GSC loss phenotype. However, we are not able to examine Stat92E in *tefu* knockdown testis as these larval gonads do not contain any germ cells, suggesting that tefu functions early in development for the formation of GSCs. As discussed in previous concern (#8), our new results show that induced DSBs do not play a major role in the maintenance of male GSCs (Fig. 6 and S7). Our results show that dRTEL1 acts through multiple downstream pathway including expression of target genes and the DDR pathway to maintain fly malel GSCs.**

10. I wonder if overexpression of dETEL1 target genes would recover the defect of dETEL1 that gives STAT92E reduction and GSCs loss. Did the authors perform any experiments addressing this question? Alternatively, any argument could be provided for this concern.

**We are sorry that we have not conducted these rescue experiments due to lack of transgenic stocks to overexpress these genes. Our results indicate that dRTEL1 may act through multiple downstream targets to regulate Stat92E level (Fig. 8N). Some of these candidates also affect Stat92E level (Fig. S6Q-T). It is possible that restoring single gene in *dRTEL1* mutant might not be able to rescue Stat92E level although we have not tested this.**

11. L405-409; germline knockdown of most of the target genes except for Nipped-A in female exhibited no GSCs reduction. Knockdown efficiency should be addressed and shown.

**We thank this reviewer for this excellent point. After performing qPCR experiments to address the knockdown efficiency in female ovary, our results show that 8 out 9 genes is significantly downregulated by its respective dsRNA construct. We also found one dsRNA construct (against *ckn*) does not reduce ckn expression in the female germline. We have now added these knockdown efficiency in Fig. S8I and removed the result of ckn. Our additional results show that expression of all these genes is not altered in ovary with germline specific knockdown of *dRTEL1* (Fig. S8J), in line with our conclusion that dRTEL1 likely does not functions through these genes to promote the maintenance of ovarian GSCs.**

12. Among the 654 target genes, 22 down-regulated genes and 49 up-regulated genes were identified. The criteria for the target genes should be described in the manuscript. In addition, I wonder whether the fold enrichment of ChIP binding has any relevance to the transcriptional level in RNA-seq data?

**The criteria for target genes have now been described in “Chromatin immunoprecipitation sequencing (ChIP) and data analysis” in “Materials and Methods” (page 41-42). We didn’t find significant correlation between the fold enrichment of ChIP binding and the transcriptional level in RNA-seq data.**

13. Line 616: ‘P-values and data significance was calculated according to two-tailed Student’s t-test.’ Where are these values shown? Although P-value is shown only for Nipped-A in text line 269, significance is not shown in Fig 5B.

**We thank this reviewer for pointing this out and now have revised Fig. 5C (Fig. 5B in previous submission) with statistical analysis.**

14. Line 715: RNA-seq samples were prepared from ALH 60-72hr testes, while DNA samples for ChIP-seq were prepared from the adult testes. What is the reason why the different developmental stages were used? The reason can be mentioned in the main text.

***dRTEL1* locates on X-chromosome and its mutant is pupal lethal, so no adult survive. Furthermore, *dRTEL1* larvae exhbit progressive male GSC loss from ALH 48hr to ALH 96hr. Thus, we collected RNA seq samples from ALH 60-72hr to minimize the effect of GSC loss on the representive cell population (see text at page 12, line 233-235, and Fig S3A-B). For ChIP-seq experiment, it is technically challenging to collect larval samples (see below). Our data show that dRTEL1 also functions in adult stage to maintain male GSCs (Fig. 2), suggesting that dRTEL1 likely functions through same set of downstream targets to maintain male GSCs at larval and adult stage. We have now added these information in the text (page 8-9)**

**For each ChIP experiment, ~800 males were dissected and testes were pooled together as one sample. For L3 testis, it requires more than 1,000 pairs of larvae testes (according to our experience) to get similar amout of material for one experiment.**

15. Fig5C: the panel shows only one input. There may be another input sample to show.

**We thank this reviewer for pointing this out and have now added another input in Fig. 5B (Fig. 5C in previous submission).**

Minor points:

1. Gal4 drivers should not be omitted in labels of the Figures; they should be written as tj-Gal4, nos-Gal4, and so on.

**We thank this reviewer for his/her suggestion and have now re-labelled the Gal4 drivers in relevant panels.**

2. Fig2DE; With, Vasa signal seems to be reduced in Tj-Gal4> dRTEL1dsRNA, but not in bam-Gal4 > dRTEL1dsRNA. I wonder if somatic dRTEL1 knockdown have some effect on the germ cell viability or gene expression. Fig2H; ‘dRTEL1 shRNA’ should be ‘dsRNA’?

**We thank this reviewer for this concern. We carefully checked more images of *Tj-Gal4 > dRTEL1 dsRNA* and didn’t find noticeable changes of Vasa signals between *Tj-Gal4 > dRTEL1 dsRNA* and *Tj-Gal4 > GFP dsRNA*. The slightly difference in Vasa signal is due to the different section plane during confocal imageing.**

**For “dRTEL1 shRNA”, we appreciate this review for pointing out our mistake and have now corrected this labeling (Fig2H).**

3. FigS3C: Describe the number of replicates and the standard deviations. Fig S3C legend: “same difference” > “some variations” ?

**We thank this reviewer for pointing this out and have now revised this accordingly (now in Fig. S3E).**

4. Line 257: ‘14.5% of the enrichment located within a 2-kb region upstream or downstream of gene coding region and 72.5% of the enrichment detected in the gene body region (Fig. 5A)’ How was this analysis performed? Describe the process in the text or method.

**We have now added this description in “Materials and Methods” as fellow:**

**"****Peaks were classified based on the location (UCSC annotation data) and showed in the following genome regions: intergenic, introns, downstream, upstream and exons”. We have also further elaborated the result as “that 11.8% of dRTEL1 enrichment located within a 2-kb region upstream (5.8%) or downstream (6%) of the gene coding region, 15.9% of the enrichment detected in the coding region and 67.5% in the intronic region” (see page 14, line 276-280).**

5. Fig4; label the specific gene names on it.

**We initially planned to label the specific gene names on Fig4. However, There are 1817, 1817, 136, 38 and 16 genes in Fig 4A,B, C, D and E and it’s difficult to label all gene names on Figures. So we state this point in the manu and add the detail imformation in Table S1, S2 and S3.**

6. 9 down-regulated genes were identified by ChIP are essential in GSCs maintenance. Though qPCR shows their expression in testes, I wonder their expression patterns in testes—are they stage or cell-type specific? Have the authors examined those?

**We thank this reviewer for this comment. To address this concern, we have now conducted RNA in situ experiment for one candidate Nipped-A to examine its expression in testis and ovary. As shown in Figure appended below, Nipped-A transcripts are detected in both germline and somatic cells, similar to that of dRTEL1. Furthermore, our new data show that male germline-specific knockdown of dRTEL1 results in downregulation of these candidate genes (Fig. S4E), indicating that they express in the male germline and this is in line with the results that germline knockdown of these candidates lead to a GSC loss (Fig. 5D-5F, S4D, and Table S5).**

**Figure. Nipped-A in situ.** (A-A’) Representative image of *Nipped-A* sense probe which does not detect specific signal in WT testis. (B-B’) Representative image of WT testis showing *Nipped-A* transcripts detected by anti-sense probe (indicated by arrows). (C-C’) Representative image of WT germarium showing *Nipped-A* transcripts detected by anti-sense probe indicated by arrows. Scale bars: 5 μm.

7. Check the Fig numbers are correct. L301: S5F and S5G>S5F-H?

**We thank this reviewer for pointing this out, the Fig numbers are now Fig. 7G-H, 7K, 7L-M and S6A-B.**

8. L386-388; ‘The Drosophila ovarian GSCs are in direct contact with cap cells, contain an anteriorly-positioned spectrosome and express pMad.’ The ovarian GSC marker, pMad, should be properly introduced.

**We thank this reviewer for this suggestions and have now added pMad information in “Introduction” (page 5, line 76-80) as follow,**

***In addition to this, the niche also provides Dpp (Decapentaplegic) and Gbb (glass bottom boat), two fly BMP ligands, which bind the receptors to activate downstream Dpp/BMP signaling in GSCs by converting Mad (mothers against Dpp) to pMad (phosphorylated Mad) which subsequently enters nucleus to repress transcription of the differentiation-promoting factor bag of marbles, bam.***

9. Fig7Q was not stated in the legend.

**We have now added this in the legend accordingly (Fig. 8N in this submission).**

10. L407 does not correspond to the figures and genes. Fig8K is for Nipped-A.

**We thank this reviewer for this suggestions, Fig. 9L (Fig. 8K in previous submission) is the control for Fig. 9M (Fig. 8L in previous submission) and Fig. S8G-Q (Fig. S6G-H in previous submission).**

11. Describe how the STAT92E intensity was quantified in Fig8L-O to induce Fig 8P.

**We think reviewer refers to the Stat92E intensity in Fig7L-O and 7P (Fig 8A-B and 8K-L and Fig 8C in current submission) and have now described it in “Materials and Methods” (page 34), as fellow:**

***The relative fluorescent Intensity of proteins in GSC were measured by FIJI (Image J).***

12. S6L; p53 signal is faint. A better image should be provided.

**We have now replaced it with a new image (Fig. S8K).**

13. Fig5A; percentiles of each category should be provided in the figure.

**We thank this reviewer for pointing this out and have now added percentiles of each category in Fig 5A.**

14. Fig5C: Add the Y-axis label. Is this a log scale? What does the arrow mean at the top of this panel? Is it a peak summit? If so, show the peak region, too.

**We thank this reviewer for pointing this out. The y-axis represents normalized read density in reads per million (rpm). We have now added this in figure legend. The peak summit was indicted in Fig. 5B by black arrow.**

15. Line 704: ‘using RESM software42’ Is this a correct description?

**We are sorry for our mistake and have now revised it as “using a software called RESM.”**

16. Line 717: ‘Two replicates were generated for control or experiment group.’ What is the control sample here? Is it the input DNA that is subjected to IP?

**We are sorry for the ambiguous decription here. The control sample is the chromatin immune-precipitated by a ChIP-grade lgg antibody (ab171870, Abcam). We have now added this description in “Materials and Methods” part.**

17. Line 721: ‘The chromatin was immune-precipitated by a ChIP-grade GFP antibody (ab290, Abcam). Sonication was conducted on Bioruptor sonicator’ Sonication must have been done before IP.

**We are sorry for the ambiguous decription here and have now clarified this in methods section as fellow:**

***“Sonication was conducted on Bioruptor sonicator (diagenode) for 30 second on-off pulse, 30 cycles at 4°C. The fragment size of sonicated chromatin was confirmed between 200bp to 500bp. The chromatin was immune-precipitated by a ChIP-grade GFP antibody (ab290, Abcam).”***

18. Fig4B: Add scale value of log2 fold change.

**We thank this reviewer for pointing this out and have now added scale value of log2 fold change in Fig 4B.**

19. Table S3 lists 140 genes as “Genes identified to be required for germline development in Sanchez et al., 2016.” However, the text (Line 237) and Figure S3D describe that 136 genes are common between this study and previous study (). Why are those numbers different? Similarly, 41 genes were listed in but 38 genes were stated in text and Figure S3D.

**We are sorry for the inaccurate description in previous submission. Those 140 genes listed in Table S3 includes 136 down-regulated genes and 4 up-regulated genes in Sanchez et al., 2016. Those 41 genes in Table S3 included 38 down-regulated genes and 3 up-regulated genes in Yu et al.,2016. We have now added these information in Excel files.**

Reviewer #3: (note: The reviewer accidentally pasted these into the Comments to Editor box instead of the Comments to Reviewer box so you may not see them online)

In this work, Yang and colleagues describe the role of dRTEL1 in the germline stem cells (GSCs) of the Drosophila testis (and ovary). The loss of GSCs in dRTEL1 mutants is clear and convincing, although clonal analysis should be done to better analyze the mutant phenotype, in particular their claim that loss of dRTEL1 induces premature GSC differentiation. In other experiments, they demonstrate that dRTEL1, which is involved in DNA repair and telomere maintenance in other systems, does indeed function through this pathway in the germline. My main concern is that dRTEL1 appears to be a general, conserved regulator of DNA repair and is likely doing this in most or all tissues of the fly. Thus, this is not a specific regulatory mechanism found in GSCs. It is not so surprising that blocking an essential cellular function like DNA repair would be detrimental for the cells in which it is blocked. It is a bit like saying that other factors essential for basic cellular processes, such as RNA polymerase or a cytoskeletal protein, are important in a particular cell type. It is nice to demonstrate this, but no one really doubted that such basic cell biology is important in most cells, and we don’t really learn anything new about the biology of that specific cell type. I feel that, once certain experiments like the clonal analysis are done, a subset of this work would be suitable for a more specialized journal than PLOS Genetics.

**We appreciate this reviewer for his/her criticism and constructive comments, which is very helpful to improve our manuscript. We have now carried out additional experiments to address these concerns and our data show that dRTEL1 has a role in the maintenance of GSCs in addition to its role in suppressing DSBs (presumably the downstream DSB repair pathway).**

**Firstly, While knockdown of dRTEL1 using nos-Gal4 in male GSCs produces a GSC loss phenotype (via differentiation not cell death or arrest), its knockdown in CySCs (the somatic stem cells which promote GSC self-renewal and maitenance) by Tj-Gal4 does not lead to a CySC loss phenotype (Fig. 2D-E). Additionally, knockdown of dRTEL1 in differentiating spermatocyte using bam-Gal4 also does not produce noticeable germline defects (Fig. 2I-J). These data suggest that dRTEL1 acts in the GSCs but not in CySCs (somatic cyst stem cells) or differentiating germline cysts to promote its maintenance. Furthermore, somatic ECs in ovary (escort cells that promote ovarian GSC maintenance and differentiation) are also maintained with somatic knockdown of dRTEL1 using tj-Gal4 (Fig. S8E-F and not shown). These data suggest dRTEL1 might have context-dependent function.**

**Secondly, among its well-established roles (telomere maintenance and double strand breaks, DSBs), RTEL1 plays important roles in DNA repair by preventing the formation of G4/R-Loops (Barber L et al., Cell 2008, Vannier JB et al., Cell 2012, Kotsantis P et al., Cell Rep 2020, We W et al., Nat Struc Mol Biol 2020, Takedachi A et al., Nat Struc Mol Biol 2020). One recent publication shows that loss of RTEL1 in mouse embryonic fibroblast (MEFs) results in an extensive transcriptional alteration. It further demonstrates that MEFs treated with G4/R-Loops stabilizer drug (10 µM) TMPyP4 results in smililar transcriptional changes, confirming that dRTEL1 suppresses G4/R-Loops formation to prevent DSBs and avert replication-transcription collisions (Kotsantis P et al., Cell Rep 2020). Consistently, our new data show that *Drosophila* larvae fed with various concentration (1 µM , 10 µM and 50 µM ) of TMPyP4 exhibits extensive DSBs in GSCs, similar to that in dRTEL1 mutant or knockdown testis (Fig. 6A-D). However, these larvae (even under higher concentration of 50 µM TMPyP4) develop into adults and these adult GSCs also exhibit elevated DSBs (Fig. 6F-I). Surprisingly, both larval gonads and adult testes maintain their GSCs and donot exhibit a GSC loss. These data show that male GSCs can be maintained in the presence of elevated DSBs induced by G4/R-Loops formation and strongly suggest that ectopic DSBs in dRTEL1 mutant or germline-specific knockdown is not the main cause of the observed GSC loss and dRTEL1 has a DSB-independent activity for GSC maintenance. Supporting this, we identify several dRTEL1 downstream candidates, whose germline-specific knockdown lead to a GSC loss (Fig. 5F). Collectively, our results show that dRTEL1 functions through multiple downstream paths (including those candidate genes and the DSB repair pathway) to promote maintenance of male GSCs. Supporting the independent/parrallel relation between these candidate genes and the DSB repair pathway, our further data show that 1) TMPyP4 treatments (on S2 cells and larvae/adult testes) do not alter expression of these candidate genes (Fig.S5) and, 2) germline-specific knockdown of these candidate genes do not produce ectopic DSBs in GSCs (Fig. S6J-N).**

**Thirdly, following data suggest that dRTEL1 affects expression of those target genes independent of DSBs and might be context dependent. First, these target genes are downregulated in both dRTEL1 mutant and testis with germline-specific knockdown of dRTEL1. Whilst their expression is not altered in S2 cells-treated with DSB-inducer TMPyP4 or testis of larvae or adult fed with TMPyP4. Additionaly, while dRTEL1 affects expression of these candidate genes in male germline, ovary with germline-specific knockdown of dRTEL1 does not exhibit altered expression in these candidate genes. Lastly, ovary of female fed with TMPyP4 also does not exhibit transcriptional change of these target genes.**

Other experiments in the paper are more problematic in my opinon. They conduct RNA-seq comparing wt to dRTEL mutant larval testes. However, they clearly document that the germline in dRTEL mutant testes is strongly reduced. Thus, this experiment is more likely to identify genes that are specific for the germline rather than genes altered more specifically by loss of dRTEL itself. They then go on to conduct CHIP-seq analysis with a tagged form of dRTEL. First of all, there is no evidence that this DNA repair protein can act as a transcription factor. Thus, CHIP-seq seems misguided at this stage. In addition, they claim a significant overlap between the RNA-seq and CHIP-seq analysis. However, while the RNA-seq analysis identified 14.9% of all genes as different between wt and dRTEL mutants, only 10.8 % of their CHIP-seq hits were also identified in the RNA-seq. Thus, there is actually a selection AGAINST the CHIP-seq targets in the RNA-seq data. Regardless of all of this, there is no evidence that suggests that dRTEL does anything other than function as a general factor in DNA repair. While the molecular mechanisms of DNA repair are certainly important to study, the fact that DNA repair is an important process for the germline, as it is for other cell types, is not significant enough on its own for publication in PLOS Genetics.

**Again, we thanks this reviewer for the insightful comments. Regarding the materials for RNA-seq, we thank this reviewer for pointing this out and we are sorry that we only briefly stated this in “Materials and Methods” and did not explain it explicitly in the text in our previous submission. As presented in Figure 1, dRTEL1 testis initially contains 6-8 GSCs at 48 hr ALH (Fig. 1C, 1D, and 1H), similar to WT controls. However, dRTEL1 mutant testis exhibits gradually GSC loss during development and by 96 hr ALH, mutant testis contains fewer GSCs (Fig. 1F-1H). To compare the transciptome difference between dRTEL1 mutant germ cells and WT germ cells, we collected early (60-72hr) L3 testis when dRTEL1 testes still contained many GSCs and GBs, minimizing the difference between control and *dRTEL1* samples. We have now explained this in main text (page 12, line 233-235) and added the representative images in Fig. S3A-B.**

**We also agree with this reviewer that our previous verification of RNA-seq data using dRTEL1 mutant testis samples which included both germ cells and somatic cells, does not differentiate the germline vs. somatic effect of dRTEL1 loss-of-function on these candidate genes. We have now conducted new experiments by using testis samples with germline-specific knockdown of dRTEL1 (at an early time point before showing a GSC loss phenotype), which is morphologically similar to control testes, to examine expression level of those candidate genes. As shown in Fig. S4E, the new results show that expression of those 9 candidate genes identified in previous submission indeed is downregulated, confirming that dRTEL1 affects expression of these genes in the male germline. Of note, in S2 cells, knockdown of dRTEL1 only affects expression of two (CG3527 and Nipped-A) but not other seven candidate genes (Fig. S4G), indicating that dRTEL1 might affect downstream gene expression in a context-dependet manner. Consistent with this, expression of these candidate genes is not altered in ovary with germline-specific knockdown of dRTEL1 (Fig. S8J). Furthermore, ectopic germline expression of dRTEL1 in testis leads to upregulated expression of some of these targets including CG34109 and Nipped-A (Fig. S4F). Together with the recent publication showing that RTEL1 colocalizes with active RNA polymerase II engaged in transcriptional elongation stage (Takedachi A et al., Nat Struc Mol Biol 2020), it is tempting to suggest that dRTEL1 does not simply act in the DSB repair pathway and it might affect target gene expression in a RNAP II-dependent manner, directly or indirectly. Further investigation is warranted to address this.**

**We continue to appreciate this reviewer for his/her critical comments regarding the ChIP experiment and data anaylsis. It’s well documented that vertebrate RTEL1 prevents DSBs via its function to suppress formation of G4/R-Loops, presumably at gene’s promoter region (Kotsantis P et al., Cell Rep 2020). Our ChIP data provide an evidence that dRTEL1 also associates with chromatin at multiple geonmic loci. Although further study is required to address whether these binding sites possess G-quadruplex (G4) sequence which frequently locates at promoter region, our ChIP data show that dRTEL1 also enriches in the gene body. This enrichment might be in line with the publication showing that human RTEL1 colocalizes with active RNA polymerase II (RNAP II) engaged in transcriptional elongation (marked by P-Ser2 at the CTD domain). Additional experiments are required to understand the exact role of active RNAP II-associated RTEL1 and whether this association might explain how dRTEL1 affect expression of some target genes independent of DSBs (also see below).**

**We fully agree with this reviewer that there is minimal overlap in target genes between RNA-seq and ChIP-seq analyses. (We are sorry if our previous submission gave this reviewer the wrong impression that we claimed significant overlap between these two analyses, which was not our intention.) As this reviewers points out, the drastic transcriptional alteration is likely an indirect (general) effect of elevated DSBs upon loss of dRTEL1 function. Similar (extensive) transcriptional alteration has been reported when murine RETL1 is removed in mouse MEFs, which is similar to MEFs treated with DSB inducer/G4 stabilizer TMPyP4 (Kotsantis P et al., Cell Rep 2020). Analyses of RNA-seq and ChIP-seq data however allows us to narrowdown some potential targets. Our new data present in Fig. S4E show that these target genes are downregulated in testis with germline-specific knockdown of dRTEL1 but not in testis with elvated DSBs induced by G4 stabilizer TMPyP4 (Fig. S5), suggesting that dRTEL1 affects expression of these genes independent of its role in DSBs (hence the DSB repair pathway) and the role of dRTEL1 in the maintenance of male GSCs are not all attributed to the defects in DSB repair. As expected, TMPyP4 feeding induces extensive DSBs in GSCs of larvae gonad. Suprisingly, these GSCs are maintained throughout development to adult stage, showing that extensive induced DSBs are not sufficient to induce a GSC loss and hence DSBs (presumablely induced by loss of dRTEL1) do not play a dominant role in the maintenance of male GSCs, distinctively different from female GSCs which undergo premature loss by transiently induced DSBs (Ma X et al., Dev 2016). Furthermore, testes with germline knockdown of these candidate genes exhibit a GSC loss with reduced Stat92E expression but without elevated DSBs detected, indicating that these genes do not act through the DNA repair pathway to maintain male GSCs. Together, our data show that dRTEL1 acts through multiple downstream events including expression of candiate genes and the DSB repair pathway to maintain fly male GSCs.**
