# Supplementary material for: dRTEL1 is essential for the maintenance of Drosophila male germline stem cells
Source: PLoS Genet. 2021 Oct 13;17(10):e1009834. doi: 10.1371/journal.pgen.1009834 (PMC8513875; doi:10.1371/journal.pgen.1009834)
Supplement: Attachment — Submitted filename: Responses to reviewers comments.docx [file pgen.1009834.s017.docx]

**Responses to reviewer’s comments**

**(PGENETICS-D-21-00484R2)**

We thank the Editor and the Reviewers for carefully reviewing our previous manuscript and the helpful comments. We have addressed the comments provided by the reviewers, point by point. Please note that all changes to the manuscript in relation to the reviewer’s comments are purple-colored in the revised manuscript. We truly believe that your constructive comments have led to a much-improved manuscript.

**Responses to the Reviewers’ comments:**

**Reviewer’s comments:**

**Reviewer 2:**

*The authors provided responses to most of our concerns and the revised version is much improved. Additional experiments explain clearly the independence of DSB and GSC loss through dRTEL1 and the statement became more convincing. For potential publication of this manuscript in PLoS genetics, however, the points raised below (#1~5) need to be further amended and/or addressed.*

1. *The phenotype of CHK1_grp was similar to the CHK2_lok and well-presented in the revised version. Is CHL1_grp also involved in the Stat 92E activation like CHK2_lok? It will be good to provide some results or argument about this.*

**Response:** We thank the reviewer for this comment and have included the possible role of CHK1_grp in regulating Stat92E in *dRTEL1* mutant background in the revised manuscript (please refer to lines 475-477 of page 23 in the revised manuscript).

2. *mRNAs of those ‘target genes’ were reduced in the dRTEL1 mutant, and ChIP-Seq revealed dRTEL1 was bound to their loci. However, no genetic evidence was shown about the interaction of downstream ‘target genes’ and dRTEL1 in loss of GSC. The authors speculated that the multiple downstream targets would not specify the Stat92E level in their overexpression. How do authors think about this? Do those multiple downstream targets function redundantly for the GSC maintenance? Any argument could be provided for this concern.*

**Response:** Although these downstream target genes are required for the maintenance of male GSC, some of them play a role in promoting Stat92E protein levels, while others do not. These results indicate that some of these genes might act in parallel to control GSC maintenance. We have summarized this in the proposed model in the revised manuscript (please refer to Fig. 8N in the revised manuscript).

3. *dRTEL1 in female GSCs; dRTEL1 mutant exhibited GSC loss and p53 upregulation, but no reduction of expression of any target genes in females. Nevertheless, Nipped-A showed a severe phenotype in ovaries.*

3-1. *Can dRTEL1 over-expression rescue the defect of GSCs in dRTEL1 mutant female like in testes?*

**Response:** While *dRTEL1* homozygous female (*xp409/xp409;+/+* from the cross of *xp409/y;dRTEL1-GFP/+* and *xp409/FM7.GFP;+/+*) is pupal lethal like hemizygous male, introducing one copy of *dRTEL1-GFP* could fully rescue female lethality and these females (*xp409/xp409;dRTEL1-GFP/+*) are fertile with normal germline development. We have now added these data in Fig. S8R-8W in the revised manuscript (please refer to Fig. S8R-8W and lines 495-496 of page 24 in the revised manuscript).

3-2. *CHK2_lok doesn’t seem to be involved in dRTEL1 regulating GSC loss (Fig9R). In addition, most of the target genes in testes were not relevant to female GSC loss, implying a different mechanism from that in testes. Can the authors interpret or speculate about the importance of dRTEL1 function in females? With this context, it also might be better to explain the further molecular role of Nipped-A in female GSCs.*

**Response:** Our results show that *dRTEL1* mutant female ovaries exhibit a GSC loss phenotype but the remaining GSCs maintain pMad expression. These data indicate that GSC loss is not a consequence of disruption in receiving niche-derived Dpp signaling and suggest that dRTEL1 likely acts downstream of or in parallel to the Dpp signaling pathway for the maintenance of these ovarian GSCs. (please refer to lines 601-604 of page 29 in the revised manuscript).

Our results show that *Nipped-A* knockdown ovary has a different phenotype from that of *dRTEL1* mutant. While *dRTEL1* mutant exhibits a GSC loss phenotype, *Nipped-A* knockdown ovary contains 2-3 GSCs, similar to controls, but harbors extra pMad-negative spectrosome-containing cells, indicating an arrest in progeny differentiation. Thus, our data suggest that Nipped-A likely does not play a role in female GSCs.

3-3. *The focus of this study is more about the function of dRTEL1 in male GSCs maintenance, I suggest moving data addressing that in females to supplementary.*

**Response:** We appreciate this reviewer’s suggestion and have now moved the female data to supplementary Figure 8 and 9 (please refer to Fig. S8 and S9 in the revised manuscript).

4. *There is no description of amended Fig8M. Please add.*

**Response:** We appreciate this Reviewer’s suggestion and have now added description in the revised manuscript following the Reviewer’s suggestion (please refer to lines 405 of page 20 in the revised manuscript).

*We have now added this information in the text (page 8-9)*

*For each ChIP experiment, ~800 males were dissected and testes were pooled together as one sample. For L3 testis, it requires more than 1,000 pairs of larvae testes (according to our experience) to get similar amount of material for one experiment.*

*5) The above information cannot be found the revised version (page 8-9). Please check.*

**Response:** We are sorry for our mistake and have now added this information in the revised manuscript (please refer to lines 277-281 of page 14 in the revised manuscript).
